# Double Trouble: A Case Report of a Locked Knee Due to a Loose Body and a Degenerative Osteophyte

**DOI:** 10.7759/cureus.53761

**Published:** 2024-02-07

**Authors:** Muhammad Fadzil Salleh, Muhamad Karbela Reza Ramlan, Ahmad Farihan Mohd Don, Suria Hayati Md Pauzi, Badrul Akmal Hisham Md Yusoff

**Affiliations:** 1 Orthopaedics and Traumatology, Universiti Kebangsaan Malaysia Medical Centre, Kuala Lumpur, MYS; 2 Pathology, Universiti Kebangsaan Malaysia Medical Centre, Kuala Lumpur, MYS

**Keywords:** knee, arthropathy, osteophytes, intra-articular loose body, acute locked knee

## Abstract

An acutely locked knee is an incapacitating condition that requires urgent orthopedic intervention. Common causes of the locked knee include a tear of the meniscus, stump of a ruptured anterior cruciate ligament (ACL), loose body, and osteochondral injury. This report describes a rare case of an acutely locked knee due to the concurrent presence of a loose body and a degenerative osteophyte, highlighting the diagnostic challenges and treatment strategies employed for successful management. Notably, to our knowledge, there is no preceding report documenting the same etiology.

## Introduction

An acutely locked knee can be defined as sudden incapacity to attain full extension of the knee, while flexion remains unaffected. Typically, this condition arises due to mechanical obstruction resulting from intra-articular abnormalities. Common causes include a tear of the meniscus, stump of a ruptured anterior cruciate ligament (ACL), loose body, and osteochondral injury [1]. Degenerative osteophytes, on the other hand, are commonly observed in osteoarthritis and can contribute to joint pain and stiffness.

Immediate and appropriate intervention is crucial for an acutely locked knee, as an extended locked position may lead to contractures and the development of a flexion deformity. Additionally, continual weight bearing on a flexed knee can cause ineffective load distribution on the tibio-femoral articulation, thereby fostering premature cartilage degeneration.

This case report describes a unique presentation of a locked knee caused by the coexistence of a loose body and degenerative osteophyte, highlighting the diagnostic challenges and treatment strategies employed for successful management.

## Case presentation

A 61-year-old office administrator who is an active tennis player presented with a complaint of recurrent episodes of a locked left knee associated with pain and functional limitations for three years. He described the sensation of his knee as "catching and getting stuck" during movements, limiting his ability to fully extend the left knee. The locking episodes resolved spontaneously with rest and with manual manipulation of the knee. The patient reported that the episodes of locked left knee increased in frequency in the past four months and were taking longer to resolve. The patient denied any prior injury or trauma to the left knee. There was also no history of instability in either of his knees.

On examination, the patient walked with an antalgic gait with the left knee in 15-degree flexion. A local examination of the left knee revealed extension loss of 15 degrees with effusion. There was pain on further passive extension of the knee and on medial joint line palpation. Otherwise, the patient was able to achieve full flexion, and the ligament test was unremarkable. There was no quadriceps muscle wasting.

**Figure 1:**
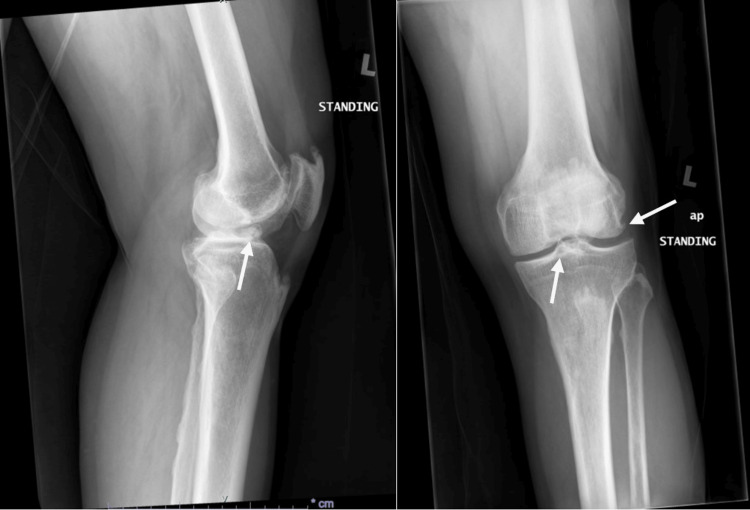
Plain radiograph showing marginal osteophytes and presence of radiopaque body over the left knee

**Figure 2:**
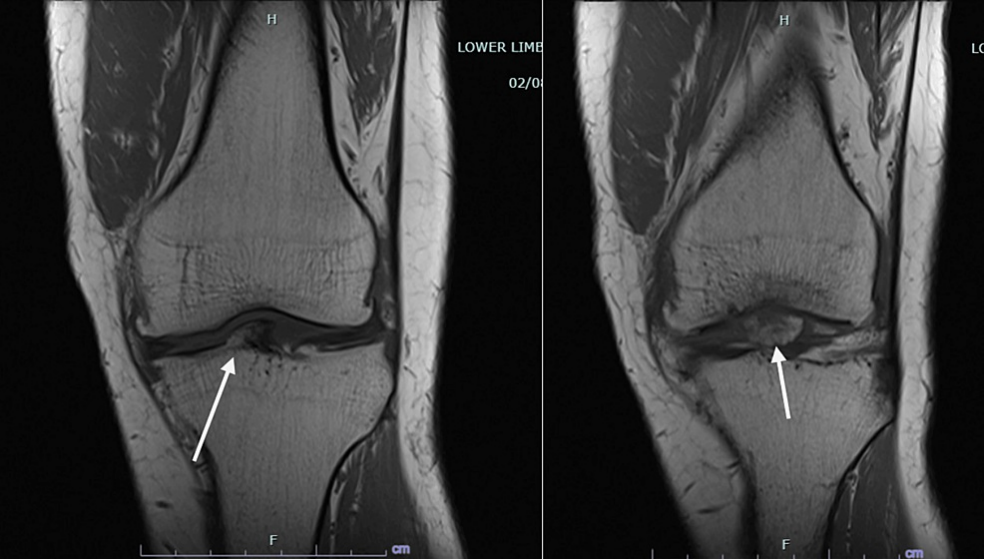
Coronal view of the MRI, demonstrating the presence of tibial osteophyte and loose body

**Figure 3:**
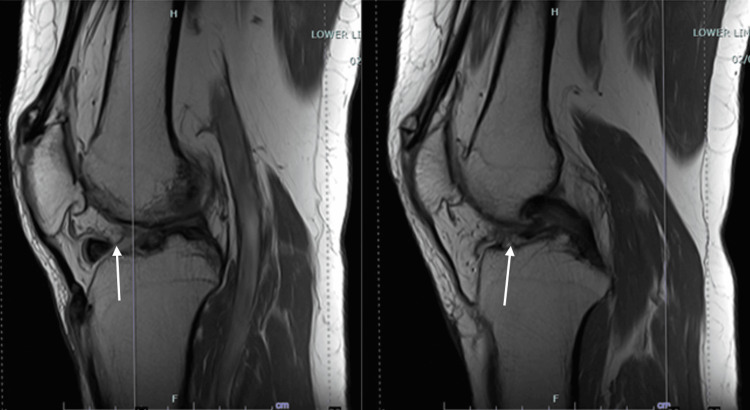
Sagittal view of the MRI, demonstrating the location of the loose body and tibial osteophyte

A plain radiograph of the left knee (Figure 1) showed joint line narrowing, multiple degenerative osteophytes, and a radiopaque body. MRI (Figure 2, Figure 3) revealed a well-defined intra-synovial structure with central isointense and peripheral hypointense rim in all sequences seen anterior to the tibial spine, likely to represent intraarticular loose bodies. There were also chondral injuries of the patella-femoral joint and both femoral condyles. The patient was then counseled for diagnostic arthroscopy of the left knee, debridement, and the removal of the loose body.

**Figure 4:**
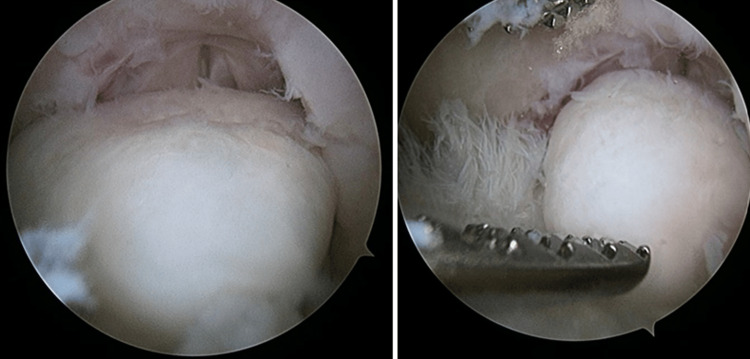
Intraarticular loose body over the femoral intercondylar notch

**Figure 5:**
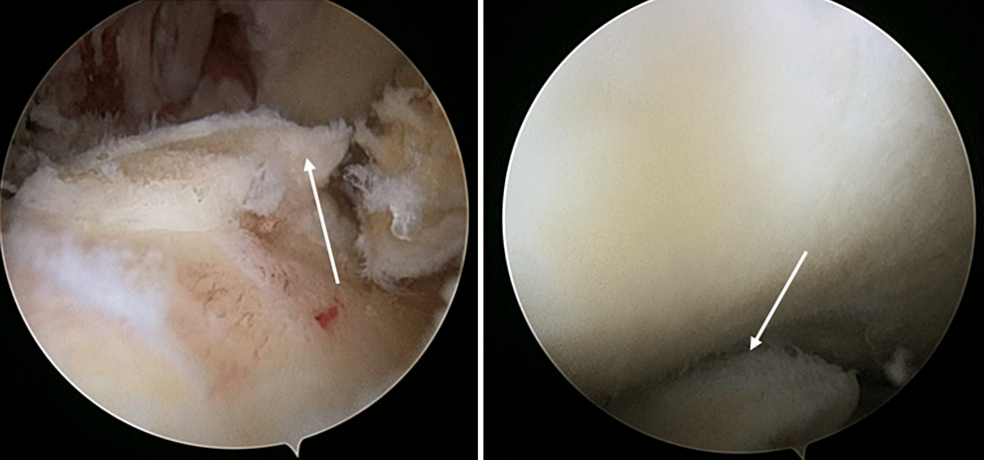
Tibial osteophytes causing mechanical blockage during extension of the left knee

**Figure 6:**
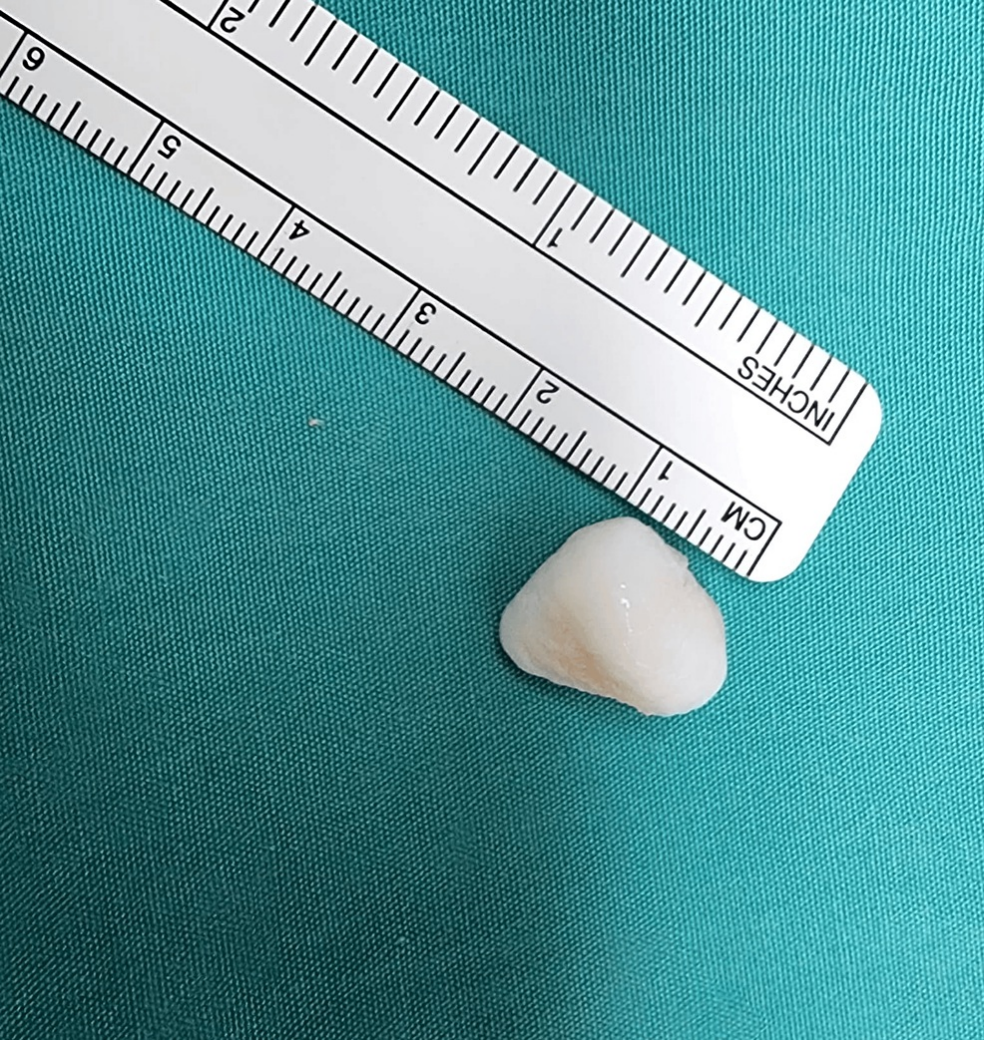
Loose body removed en-bloc arthroscopically

The left knee remained locked in 15-degree flexion under anesthesia. Intraoperative findings revealed a loose body measuring 15mm x 10mm x 5mm over the femoral intercondylar notch (Figure 4) and tibial eminence osteophyte (Figure 5) causing mechanical blockage during extension of the left knee. An arthroscopic debridement of the tibial eminence osteophyte was done, and the loose body was removed en bloc arthroscopically (Figure 6). A full range of motion of the left knee was achieved after the procedure. There were International Cartilage Repair Society (ICRS) grade II cartilage injuries over the patella-femoral joint and both femoral condyles, worse on the medial femoral condyle. No ligament or meniscus injury was observed.

**Figure 7:**
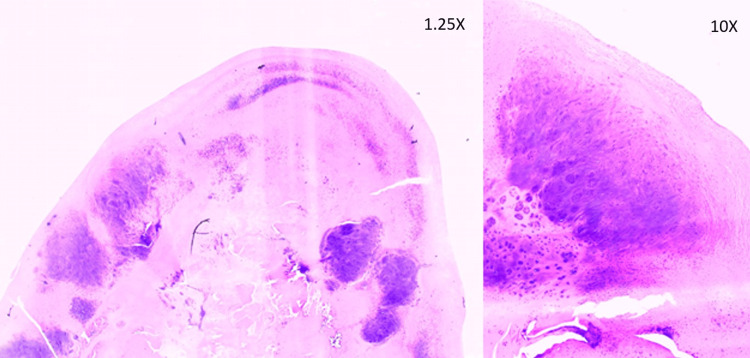
Histopathological specimen stained with H&E at 1.25X and 10X magnification showing hyaline cartilaginous tissue with fragmentation and fibrillation of matrix

The procedure was followed by a comprehensive rehabilitation program involving physical therapy, joint mobilization, muscle strengthening, and gait training. A histopathological report of the loose body showed benign cartilaginous tissue with degenerative features (Figure 7).

The patient reported complete resolution of the locking episodes and was able to achieve full range of motion over the left knee during the two weeks post-operative follow-up. He remained asymptomatic three months post-operation and reported a significant reduction in pain and improved knee function, enabling him to resume daily activities without restrictions.

## Discussion

The acutely locked knee presents as a painful and incapacitating orthopedic condition, demanding timely and appropriate intervention. It is commonly characterized by mechanical obstruction due to intra-articular abnormalities. Alternatively, the condition may also be attributed to muscle spasms secondary to ligamentous or bony injuries [2,3]. There has been one report of tibial osteophyte as an unusual cause of locked knee, highlighting the sign of osteoarthrosis as a possible cause of acutely locked knee [4]. Our case presented a concurrent presence of loose body and degenerative tibial osteophyte as the cause of this condition.

Bansal et al. [5] reported that the mechanical cause of the locked knee can be identified consistently using three key components from the history and examination: a history of a definite injury, localized joint line tenderness, and the presence of an effusion. Despite the patient denying any trauma or injury to his knee, both localized joint line tenderness and the presence of effusion can be demonstrated in our case, further raising suspicion about the mechanical nature of this condition. Given that a conclusive diagnosis cannot be reached through clinical examination alone, additional investigations are essential.

Osteophytes frequently manifest as a radiographic hallmark of osteoarthritis, typically observed along the joint margins. These formations initially emerge as cartilaginous outgrowths and undergo subsequent endochondral ossification. Osteophytes, while lacking a direct role in disease progression, function as indicators of both the location and severity of the underlying pathologic process [6]. These bony outgrowths are often reflective of the degenerative changes occurring within the joint, providing valuable insights for clinicians in assessing and understanding the extent of the pathological condition. The radiograph of our patient's left knee reveals the presence of multiple marginal osteophytes. Interestingly, the tibial eminence osteophyte is not prominently visible, likely due to overlap with the radiopaque loose body.

MRI is advised to distinguish between true-locking and pseudo-locking. True-locking involves a mechanical obstruction, while pseudo-locking is characterized by an inability to fully extend the knee due to pain or muscle spasm, not a physical blockage [7,8]. In our clinical approach, MRI is employed to identify injuries to various structures, including the cruciate ligaments, meniscus, and articular cartilage. This approach aids in preoperative planning and enables comprehensive counseling of the patient regarding the planned procedures and postoperative rehabilitation. Although MRI is a sensitive, non-invasive diagnostic test for detecting anatomic abnormalities of the knee, it is not a substitute for thorough history taking and careful physical examination.

In this case, a diagnostic arthroscopy was performed, followed by a surgical debridement of the tibial osteophyte and removal of the loose body, which was the leading cause of the patient’s symptoms. To our knowledge, this case report is the first demonstrating the concurrent presence of an intraarticular loose body and degenerative osteophyte as the cause of an acutely locked knee.

## Conclusions

In summary, although the concurrent occurrence of loose bodies and degenerative osteophytes is rare, it may be worthwhile to consider it as a differential diagnosis in cases of acutely locked knees. This case report emphasizes the significance of a comprehensive history and physical examination, diagnostic workup including imaging studies, and the effectiveness of arthroscopic management combined with structured rehabilitation, which contributed to favorable outcomes.
